# Expression of Toll-like receptor 2 is up-regulated in monocytes from patients with chronic obstructive pulmonary disease

**DOI:** 10.1186/1465-9921-7-64

**Published:** 2006-04-10

**Authors:** Jaume Pons, Jaume Sauleda, Verónica Regueiro, Carmen Santos, Meritxell López, Joana Ferrer, Alvar GN Agustí, José A Bengoechea

**Affiliations:** 1 Unidad de Investigación, Hospital Son Dureta, Institut Universitari d'Investigacions en Ciències de la Salut (IUNICS), Palma Mallorca, Spain; 2 Program Infection and Immunity, Fundació Caubet-CIMERA Illes Balears, Bunyola, Spain; 3 Servicio de Neumología, Hospital Son Dureta, Palma Mallorca, Spain; 4 Servicio de Inmunología, Hospital Son Dureta, Palma Mallorca, Spain

## Abstract

**Background:**

Chronic obstructive pulmonary disease (COPD) is characterised by pulmonary and systemic inflammation which flare-up during episodes of acute exacerbation (AECOPD). Given the role of Toll-like receptors (TLRs) in the induction of inflammatory responses we investigated the involvement of TLRs in COPD pathogenesis.

**Methods:**

The expression of TLR-2, TLR-4 and CD14 in monocytes was analyzed by flow cytometry. To study the functional responses of these receptors, monocytes were stimulated with peptidoglycan or lipopolysaccharide and the amounts of TNFα and IL-6 secreted were determined by ELISA.

**Results:**

We found that the expression of TLR-2 was up-regulated in peripheral blood monocytes from COPD patients, either clinically stable or during AECOPD, as compared to never smokers or smokers with normal lung function. Upon stimulation with TLR-2 ligand monocytes from COPD patients secreted increased amounts of cytokines than similarly stimulated monocytes from never smokers and smokers. In contrast, the expressions of TLR-4 and CD14 were not significantly different between groups, and the response to lipopolysaccharide (a TLR-4 ligand) stimulation was not significantly different either. At discharge from hospital TLR-2 expression was down-regulated in peripheral blood monocytes from AECOPD patients. This could be due to the treatment with systemic steroids because, *in vitro*, steroids down-regulated TLR-2 expression in a dose-dependent manner. Finally, we demonstrated that IL-6, whose plasma levels are elevated in patients, up-regulated *in vitro *TLR-2 expression in monocytes from never smokers.

**Conclusion:**

Our results reveal abnormalities in TLRs expression in COPD patients and highlight its potential relationship with systemic inflammation in these patients.

## Background

Chronic obstructive pulmonary disease (COPD) is characterised by an abnormal inflammatory response of the lungs to noxious particles or gases, primarily cigarette smoking, albeit not all smokers develop the disease [1]. COPD is also associated with systemic inflammation [2], which is likely to contribute significantly to some important extra-pulmonary consequences of COPD [1], namely cardiovascular disease [3] and cachexia [4,5]. Both, pulmonary and systemic inflammation, flare-up during the episodes of acute exacerbation (AECOPD) that occur often in these patients [1]. It is generally accepted that some form of bacterial and/or viral infection is the main cause of AECOPD [6], but the precise molecular mechanisms underlying these episodes have not been fully characterized [7]. Further, the relationship between bacterial airway colonization and the abnormal pulmonary and systemic inflammatory response that characterizes COPD is unclear.

Mammalian Toll-like receptors (TLR) comprise a family of germ line-encoded trans-membrane receptors which recognize conserved microbial structures, the so called pathogen-associated molecular patterns (PAMPs) [8]. Activation of TLRs leads to the induction of inflammatory responses and to the development of antigen specific adaptive immunity [8,9]. Among this family of receptors, TLR-2 and TLR-4 have received great attention. TLR-4 is essential for the recognition of lipopolysaccharide (LPS), a major component of Gram-negative bacteria, whereas TLR-2 recognizes a large number of ligands including bacterial lipotheicoid acid and lipoproteins [8]. CD14 is a 55-kDa GPI-linked glycoprotein that also participates in pathogen recognition and uses TLRs as co-receptors in signal transduction [10]. It has been shown that microbial components interact primarily with CD14 and subsequently with the TLRs [11].

Because many patients with stable COPD present airway colonization [12,13] and bacterial infection is a key trigger of AECOPD [6], we hypothesized that TLR may participate in the regulation of inflammation in COPD, particularly during the episodes of AECOPD. To investigate it, we first compared the expression of TLR-2, TLR-4 and CD14 in circulating monocytes harvested from COPD patients (both during AECOPD and when clinically stable), smokers with normal lung function and never smokers. Then, we investigated the functionality of these receptors upon stimulation with specific ligands. Finally, we studied the effect of steroids, a drug routinely used in the treatment of AECOPD [1], and IL-6, a cytokine known to be elevated in the systemic circulation of COPD patients [2], upon the expression of TLR-2, TLR-4 and CD14.

## Methods

### Population and ethics

All participants gave their written consent after being fully informed of the study, which was previously approved by the Ethics Committee of our institution. Patients with COPD were considered clinically stable if they had not had an AECOPD episode and/or had required a change in their usual therapy during the last 3 months. COPD patients were treated with long-acting inhaled bronchodilators and 6 received inhaled steroids but none was under oral steroid therapy. Subjects with atopic diseases, allergic rhinitis and asthma were excluded. To avoid any potential effect of acute smoking, active smokers refrained from smoking 12 hours before examination; exhaled carbon monoxide concentration was lower than 10 ppm in all subjects. Patients with AECOPD were studied within the first 24 hours of hospital admission; at discharge and 3 months later, when clinically stable again. Healthy subjects and smokers with normal lung function were recruited from the pulmonary function laboratory of our institution.

Because of the small volume of blood collected (5 to 8 ml), we could not perform all the analysis for each patient and therefore the cell stimulation experiments were performed only with a small group of them. Indeed, performing all the experiments with the cells of the same patient was not allowed by the Ethics Committee because of the large blood volume (30 ml) needed and since many of them was hospitalized due to a worsening of their clinical status.

### Bacterial isolation

AECOPD and COPD patients spontaneously expectorated sputum samples when the clinics visit. Samples were homogenized, diluted, and plated for identification as previously described [6,14]. Patients did not receive antibiotics prior to sputum cultures.

### Lung function

Forced spirometry (GS, Warren E. Collins, Braintree, MA, USA) was obtained in all participants according to international guidelines [15]. Spirometric reference values were those of a Mediterranean population [16].

### Purification of peripheral blood monocytes

Peripheral blood mononuclear cells were purified by centrifugation on Ficoll-Hypaque, and monocytes were obtained using a commercial isolation kit exactly as recommended by the manufacturer (Dynal monocyte negative isolation kit, Oxoid). Lymphocytes represented less than 5% of the cells after this procedure. Cells were finally resuspended at a cell density of 10^6 ^cells/ml in RPMI-1640 medium supplemented with 10% heat inactivated Fetal Calf Serum (FCS), glutamine (2 mM), HEPES (200 mM) and antibiotics (penicillin-streptomycin). These purified monocytes were used for the experiments shown in figures 2, 4 and 5.

**Figure 1:**
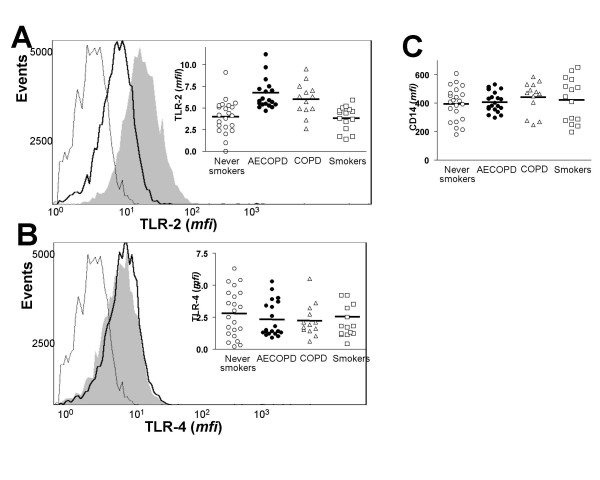
Analysis of the expression of TLR-2 (panel A), TLR-4 (panel B) and CD14 (panel C) in peripheral blood monocytes from never smokers (n = 22), AECOPD (n = 20) and COPD (n = 13) patients and smokers (n = 20). Shaded area represents TLR staining of monocytes from a representative AECOPD patient. The un-shaded area outlined by the darker line represents TLR staining in monocytes from a representative never smoker. The un-shaded area outlined by a thin line represents isotype matched PE labelled antibodies staining in monocytes from the AECOPD patient. The results were analyzed by one-way analysis of variance with Bonferroni contrasts.

**Figure 2:**
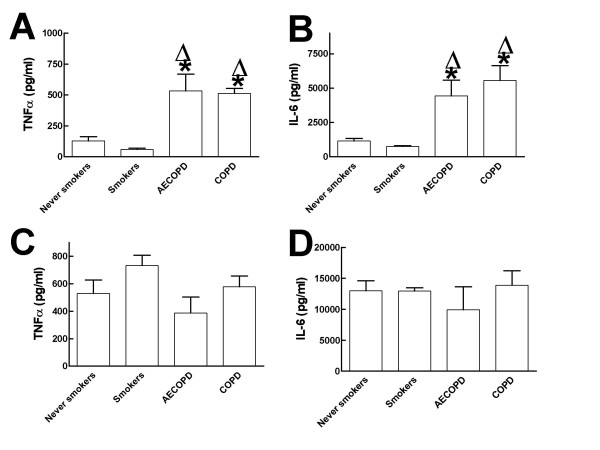
Levels of TNFα and IL-6 secreted into culture medium by purified monocytes from never smokers (5 subjects; purified cells from each subject were tested in triplicate), smokers (5 subjects; purified cells from each subject were tested in triplicate), AECOPD patients (AECOPD, 5 subjects; purified cells from each subject were tested in triplicate) and COPD patients (COPD, 5 subjects; purified cells from each subject were tested in triplicate). Monocytes were stimulated with 1 μg/ml of peptydoglycan (PGN) and supernatants were analyzed for TNFα(panel A) or IL-6 (panel B). Monocytes were stimulated with 100 ng/ml of LPS and supernatants were analyzed for TNFα(panel C) or IL-6 (panel D). The results were analyzed by one-way analysis of variance with Bonferroni contrasts. Symbols: * significant difference (p < 0.05) versus never smokers; Δ significant difference (p < 0.05) versus smokers.

**Figure 3:**
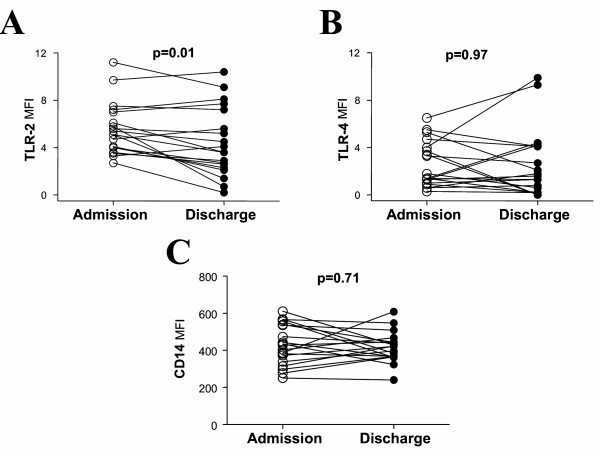
Analysis of TLR-2 (panel A), TLR-4 (panel B) and CD14 (panel C) expression in peripheral blood monocytes from AECOPD patients at admission (open circles) and hospital discharge (black circle). The results were analyzed by paired two-tailed t test.

**Figure 4:**
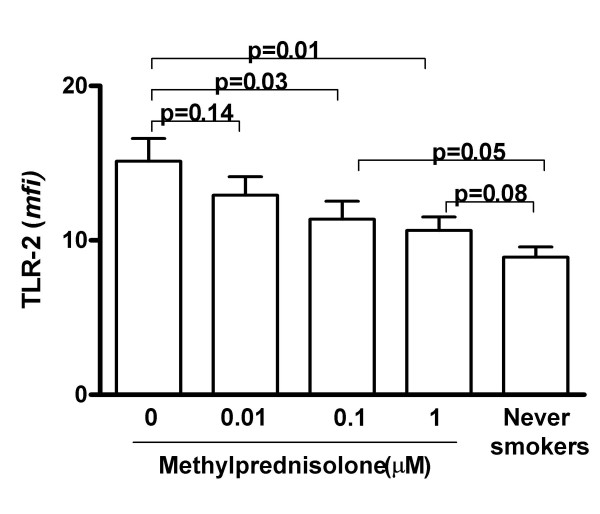
Effect of methylprednisolone on the expression of TLR-2 by monocytes from AECOPD patients (5 different patients; purified cells from each subject were tested in duplicate for each condition studied). Purified monocytes were incubated for 3 h with different amounts of methylprednisolone and TLR-2 expression was studied by flow cytometry. For statistical comparisons, TLR-2 expression by monocytes from 5 representative never smokers after 3 h culture without stimuli is included in the figure. The results were analyzed by one-way analysis of variance with Bonferroni contrasts.

**Figure 5:**
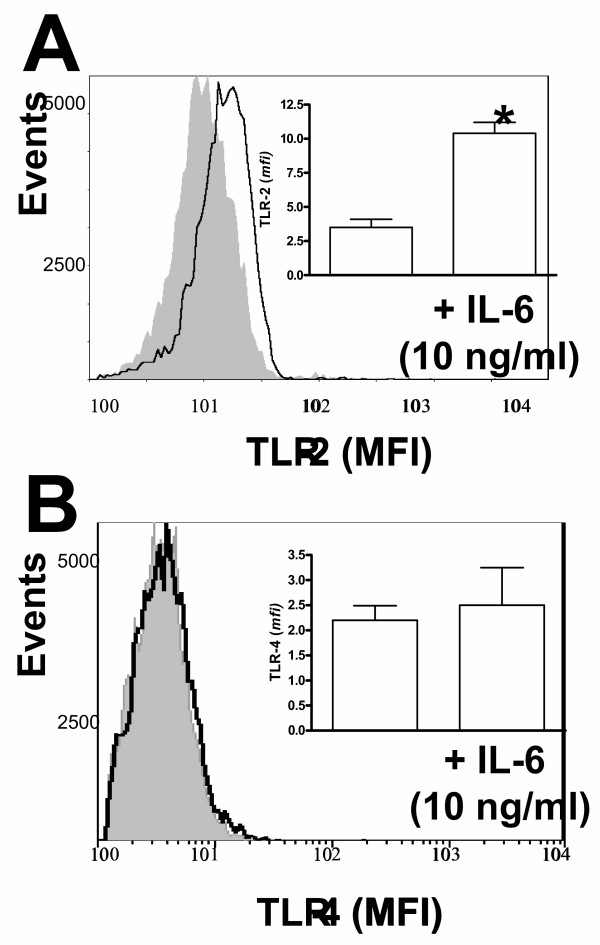
Effect of IL-6 on the expression of TLR-2 (panel A) and TLR-4 (panel B) by human monocytes from never smoker. Purified monocytes were incubated in the presence or absence of IL-6 (10 ng/ml) and 3 h later TLRs expression was analyzed by flow cytometry. Shaded area represents TLRs staining of monocytes incubated without IL-6 whereas un-shaded area represents TLRs staining of monocytes incubated with IL-6. *mfi *values of cells incubated with isotype matched antibodies were 4.3 ± 1.6 whereas *mfi *values of IL-6-treated cells incubated with isotype matched antibodies were 5.4 ± 1.2 (p > 0.05). Inset shows results of three different never smokers tested in duplicate for each condition studied. The results were analyzed by paired two-tailed t test. Symbol: * significant difference (p < 0.05) versus non-treated monocytes.

### Flow cytometry

Expression of CD14, TLR-2 and TLR-4 in peripheral blood monocytes was determined by flow cytometry. Blood samples (one sample per patient) were collected by peripheral venipuncture and incubated during 30 minutes at 4°C with a combination of anti-CD14 FITC conjugated (clone My4, 10 μg/ml; Beckman Coulter) and anti-TLR-2 (clone TL2.1, 10 μg/ml; ebioscience) or anti-TLR-4 (clone HTA125, 10 μg/ml; ebioscience) PE conjugated. Monocytes were identified by gating on a side versus CD14 dot plot.

Expression of CD14, TLR-2 and TLR-4 in purified monocytes treated with IL-6 or steroids (results shown in figures 4 and 5) was also determined by flow cytometry. Monocytes were detached from the wells with a rubber policeman, washed with 0.1 % sodium-azide in PBS and incubated with the antibodies exactly as indicated before.

The analyses were carried out in an Epics XL flow cytometer using the Expo32 software. The levels of CD14, TLR-2, TLR-4 were expressed as mean fluorescence intensity (*mfi*) measured in arbitrary units and the non specific binding was corrected by subtraction of *mfi *values corresponding to isotype matched antibodies. A minimum of 3500 monocytes were analyzed in every experiment.

### Cell culture and stimulation

Cells were cultured in 96 well plates at a cell density of 10^5 ^per well. Cells were stimulated with 100 ng/ml of lipopolysaccharide (LPS) purified from *Escherichia coli *O111:B4 (Sigma Chemicals). This LPS was repurified exactly as previously described [17]. This procedure results in enterobacterial LPS preparations that utilize TLR-4, and not TLR-2, for signalling [17]. Cells were also stimulated with 1 μg/ml of peptydoglycan (PGN) purified from *Staphylococcus aureus *(Merck). This PGN preparation does not stimulate stably transfected TLR4-MD2-CD14 HEK293 cells (data not shown) and it is also a poor activator of the intracellular receptor NOD2 [18]. Recently it has been shown that the commercial PGN preparation used in this work contains lipoteichoid acid which is the true TLR-2 agonist [18]. Perusal of the literature shows that the concentrations of TLR agonists used in this study optimally stimulate human monocytes (for example see [19]). After 16 hours cell culture supernatants were collected, cell debris were removed by centrifugation, and samples were frozen at -80°C until assayed

### Cytokine quantification

We determined the concentration of IL-6 and TNFα in cell culture supernatants or in plasma, using a bead array ELISA according to the instructions of the manufacturer (CBA Kit, BD Biosciences). The assay sensitivity for IL-6 was 2.5 pg/ml and for TNFα was 3.7 pg/ml.

### Statistical analysis

Results are expressed as mean ± SD. The results were analyzed by paired two-tailed t test or one-way analysis of variance with Bonferroni contrasts using GraphPad Prism software (GraphPad Sotware Inc.). A p value lower than 0.05 was considered significant.

## Results

### Clinical data

Table 1 shows the clinical and functional data of subjects included in the study. AECOPD patients were older than the other groups (Table 1). Patients with COPD had moderate-severe airflow obstruction, particularly those with AECOPD whereas, by design, lung function was normal in the other two groups of subjects studied (Table 1).

**Table 1:** Clinical and functional data of the subjects included in this study.

	**Never smokers ***(n = 22)*	**Smokers with normal lung function ***(n = 20)*	**Stable COPD Patients ***(n = 13)*	**AECOPD Patients ***(n = 20)*
**Age ***(years)*	57.8 ± 6.3	59.0 ± 1.9	60.0 ± 2.0	65.0 ± 2.0*
**Smoking history ***(pack years)*	0	43.1 ± 3.2	46.0 ± 3.0	65.0 ± 6.0
**FEV1 ***(% ref)*	95.8 ± 5.1	93.0 ± 2.5	58.0 ± 2.0+	38.0 ± 3.0†
**FEV1/FVC ***(%)*	81.8 ± 2.5	75.0 ± 1.0	56.0 ± 2.0+	46.0 ± 2.0†

* p < 0.05 (AECOPD vs stable COPD, smokers with normal lung function and never smokers)

†p < 0.01 (AECOPD vs stable COPD, smokers with normal lung function and never smokers)

+p < 0.01 (stable COPD vs smokers with normal lung function and never smokers)

### TLR expression

Figure 1 shows that, at admission, peripheral blood monocytes from AECOPD patients expressed significantly more TLR-2 than never smokers (6.78 ± 2.09 *mfi *vs. 4.01 ± 1.94 *mfi *respectively; p = 0.001) (fig 1 panel A) whereas the expression levels of TLR-4 were not significantly different (2.32 ± 1.4 *mfi *vs. 2.80 ± 1.85 *mfi*, respectively, p = 0.36) (fig 1 panel B). Bacteria were isolated from the sputum of only 4 AECOPD patients and in all cases the organism was identified as nontypable *Haemophilus influenzae*. In these subjects, the expression levels of TLR-2 (5.72 ± 0.6 *mfi*) and TLR-4 (2.3 ± 0.5 *mfi*) were not different from the other patients with AECOPD. Analysis of TLRs expression in peripheral blood monocytes from stable COPD patients revealed that TLR-2 expression was also up-regulated compared to never smokers (6.02 ± 1.9 *mfi *vs. 4.01 ± 1.94 *mfi*; p = 0.01) (fig 1 panel A) and not significantly different to that found in AECOPD patients at admission (5.94 ± 2.12 *mfi *vs 6.78 ± 2.09 *mfi *respectively; p = 0.28). TLR-4 expression (2.25 ± 1.34 *mfi*) was not significantly different to that of AECOPD (p = 0.34) or never smokers (p = 0.88) (fig 1 panel B). TLR-2 expression in smokers with normal lung function was not significantly different to that found in never smokers (3.40 ± 0.5 *mfi *vs 4.01 ± 1.94 *mfi*, respectively, p = 0.75) (fig 1 panel A) and this was also the case when the expression of TLR-4 was compared in these two groups (2.42 ± 2.14 *mfi *vs 2.80 ± 1.85 *mfi*, respectively, p = 0.71) (fig 1 panel B). However, monocytes from smokers expressed significantly less TLR-2 than monocytes from AECOPD patients (3.40 ± 0.5 *mfi *vs 6.78 ± 2.09 *mfi*, respectively, p = 0.001) and monocytes from COPD patients (3.40 ± 0.5 *mfi *vs 6.02 ± 2.09 *mfi*, respectively, p = 0.02). In contrast, TLR-4 expression was not significantly different to that of AECOPD (p = 0.71) or COPD (p = 0.64) (fig 1 panel B). Finally, monocytes from AECOPD patients expressed similar amounts of CD14 (407 ± 70.76 *mfi*) than monocytes from never smokers (395 ± 117.2 *mfi*), stable COPD patients (439 ± 116.8 *mfi*) or smokers (422 ± 154.4 *mfi*) (fig 1, panel C).

### TLR functionality

To study the functional response of TLRs, purified monocytes harvested from AECOPD patients at admission, stable COPD patients, smokers or never smokers were stimulated with PGN or highly purified LPS (stimuli that signal through TLR-2 and TLR-4 respectively) and the amounts of TNFα and IL-6 secreted taken as read-out for monocyte activation. No differences were observed in the amount of TNFα secreted by unstimulated monocytes from never smokers, smokers, AECOPD and COPD patients (20 ± 4 pg/ml, 27 ± 9 pg/ml 24 ± 8 pg/ml and 22 ± 4 pg/ml respectively). The basal secretion of IL-6 was also similar in the three groups (110 ± 10 pg/ml, 127 ± 22 pg/ml, 95 ± 8 pg/ml and 116 ± 15 pg/ml respectively). Figure 2 (panels A and B) shows that monocytes from AECOPD (n = 5) and stable COPD (n = 5) stimulated with PGN secreted significantly higher amounts of both cytokines than similarly treated monocytes obtained from never smokers (n = 5) and smokers (n = 5). Monocytes from never smokers secreted similar amounts of both cytokines than monocytes from smokers. When LPS was used as stimulus, monocytes from patients secreted similar amounts of both cytokines than monocytes obtained from never smokers or smokers (fig 2, panels C and D). These results are in agreement with the fact that TLR-2 expression, but not that of TLR-4, was up-regulated in monocytes from AECOPD and stable COPD patients. We did not find significant differences in the secretion of cytokines between monocytes harvested from AECOPD or stable COPD patients independently of the stimuli used.

### Effect of steroids on TLR expression

According to international guidelines [1], patients with AECOPD were treated during hospitalization with intravenous steroids (methylprednisolone 2 mg/Kg/day during 3 days with a progressive reduction of the drug in the following 11 days), bronchodilator (salbutamol 2.5–5 mg every 6 h) and antibiotics (levofloxacin 500 mg/day during 7–10 days or amoxicillin-clavulanic acid 875 mg/8 h during 7–10 days). In parallel, we found a significant reduction of TLR-2 expression in AECOPD patients studied at discharge (fig 3 panel A) that was no longer different from that of never smokers (5.56 ± 2.20 *mfi *vs 4.01 ± 1.94 *mfi *respectively; p = 0.11). In contrast, neither the expression of TLR-4 (fig 3 panel B) nor that of CD14 (fig 3 panel C) changed during hospitalization. In 6 of these AECOPD patients, TLR-2 expression was monitored 3 months after hospital discharge and an increase in TLR-2 expression was found (7.02 ± 1.52 *mfi*). Actually, these levels were not significantly different from those determined in AECOPD patients at admission (7.02 ± 1.52 *mfi *vs 6.78 ± 0.47 *mfi *respectively; p = 0.31), suggesting that TLR-2 downregulation is transient.

To further characterize the effect of steroids upon TLR-2 expression, monocytes harvested from patients with AECOPD (5 different patients) were incubated *in vitro *with increasing doses of methylprednisolone (3 h; 0.01 to 1 μM). We found that steroids down-regulated the expression of TLR-2 in a dose-dependent fashion (fig 4). A similar effect was seen when dexamethasone was used instead of methylprednisolone (data not shown).

### Role of systemic inflammation in TLRs expression

We found that the plasma concentration of IL-6 was significantly higher in sera from AECOPD patients (5.19 ± 1.03 pg/ml) and stable COPD patients (5.75 ± 0.86 pg/ml) than in never smokers (2.61 ± 0.13 pg/ml, p = 0.02). To investigate the functional role of IL-6 upon TLRs expression, purified monocytes from never smokers were incubated in the presence of IL-6 and TLRs expression was evaluated by flow cytometry. Figure 5 shows that IL-6 up-regulated the expression levels of TLR-2 (panel A; 10 ± 1.2 *mfi *in the presence of IL-6 versus 3.8 ± 0.9 *mfi *in absence of IL-6; p = 0.001) whereas the levels of TLR-4 (panel B; 2.5 ± 1.3 *mfi *in the presence of IL-6 versus 2.2 ± 0.5 *mfi *in the absence of IL-6; p > 0.05) and CD14 (data not shown) were unaffected. In parallel experiments we observed that neither IL-8 nor IL-1β modified TLR-2 expression (data not shown), thereby arguing against a general non-specific effect due to the incubation of monocytes with cytokines.

## Discussion

This study shows that the expression of TLR-2 was up-regulated in peripheral blood monocytes harvested from COPD patients, either when clinically stable or during an exacerbation of the disease, as compared to never smokers or smokers with normal lung function. Furthermore, upon stimulation with agonist signalling through TLR-2, monocytes from COPD patients secreted increased amounts of IL-6 and TNFα than similarly stimulated monocytes from never smokers and smokers with normal lung function. In contrast, the expressions of TLR-4 and CD14 were not significantly different between groups and the response to LPS stimulation (a TLR-4 specific ligand) was not significantly different. We also showed that at discharge, TLR-2 expression was down-regulated in peripheral blood monocytes from AECOPD patients. This could be due to the treatment with systemic steroids because, *in vitro*, steroids down-regulated TLR-2 expression in a dose-dependent manner. Finally, we demonstrated that IL-6, whose plasma levels are elevated in patients, up-regulated TLR-2 expression *in vitro *in purified monocytes from never smokers, thereby connecting the systemic inflammation that characterizes COPD and TLR-2 expression. Altogether, these findings may be relevant for a better understanding of, first, the mechanisms triggering the abnormal inflammatory response that characterizes COPD, particularly during the episodes of AECOPD, and, second, the molecular effects of some therapeutic options available to date.

TLRs are key molecules in host defence against microbial pathogens. TLRs recognize pathogen-associated molecular patterns (PAMPs) who trigger the expression of proinflammatory genes and the development of antigen specific adaptive immunity [8,9]. Most of our current knowledge of TLR signalling has emerged from studies of gene-targeted mice [8,9]. The contribution of TLR function to human disease is less advanced [20]. So far research has mainly focused on the relationship between presence of TLRs polymorphisms and susceptibility to a disease [20]. Since the available data indicate that there would not be a TLR polymorphism associated with COPD [21] in this study we analyzed whether the expression and/or functionality of TLRs was altered. We reasoned that the increased secretion of inflammatory mediators found in COPD patients could be due to an up-regulation of TLRs expression. This hypothesis was based on studies showing that macrophages overexpressing TLRs release higher amount of inflammatory mediators upon TLR engagement [22,23]. Indeed we found that TLR-2 (but not TLR-4) expression was up-regulated in monocytes of COPD patients (both when clinically stable and during AECOPD), and that these cells secreted elevated levels of inflammatory mediators upon challenge with preparations containing TLR-2 agonists (but not with LPS) (fig 2). However it could be possible that the upregulation of TLR-2 could not be the only explanation behind the increased levels of inflammatory mediators. Thus different levels of molecules of the TLR intracellular signalling pathway might also account for the increased secretion of mediators. However this possibility seems unlikely given the facts that TLR-2 only transduces the signal via the MyD88-dependent signalling pathway which is also used by TLR-4 [24] and that TLR-4 dependent responses were not affected. On the other hand, recently it has been shown that NOD2 recognizes PGN [25,26] and therefore activation of NOD2 dependent signalling pathway might also account for our results. However it is important to note that activation of this receptor requires an intracellular presentation of PGN which it is not the case in our experimental set up. Nevertheless it cannot be ruled out that function and/or expression of the elements of this signalling pathway could be altered in COPD patients. Future studies will address this issue.

TLR-2 up-regulation in peripheral blood monocytes can contribute significantly to the systemic inflammation that occurs in COPD patients [5]. Interestingly, Riordan et al [27] have reported similar findings in patients with liver cirrhosis, a disease that, like COPD, is associated with systemic inflammation. The airways of patients with stable COPD are often colonized by bacteria, and bacterial pathogens (mainly nontypable *Haemophilus influenzae *and *Streptococcus pneumoniae*) can be isolated in more than 70% of AECOPD [6]. Importantly, PAMPs of these pathogens activate inflammatory responses via TLR-2 among other TLRs [28,29]. These bacteria are highly fragile and tend to autolysis, thereby facilitating that their PAMPs reach the systemic circulation. Hence, PAMPs-mediated activation of monocytes via TLR-2 can contribute to the systemic inflammation of COPD. Likewise, TLRs also recognize endogenous ligands ("danger signals") produced by cells undergoing stress or necrosis [30,31]. Considering that COPD is characterized by considerable tissue injury [32], it is also possible that these endogenous ligands could engage TLRs and contribute to systemic inflammation even in the absence of bacterial PAMPs.

Several potential mechanisms can contribute to up regulate TLR-2 in monocytes from COPD patients. Smoking is not likely to be one of them because cells from smokers with normal lung function expressed similar amounts of TLR-2 than cells from never smokers (fig 1). Bacterial PAMPs may also contribute to TLR-2 upregulation in patients. In fact, it has been shown that the PAMPs'of *H. influenzae *up-regulate TLR-2 expression but not that of TLR-4 in eukaryotic cells [33]. Yet, sputum cultures in the majority of patients studied here were negative, although it is known that airway bacterial colonization can occur despite the negativity of sputum cultures [34]. Finally, inflammatory cytokines may also alter TLRs expression [35]. Indeed, we found that IL-6 up-regulated TLR-2 expression *in vitro *in monocytes obtained from never smokers (figure 5). Thus, it is possible that the pro-inflammatory milieu known to occur in COPD has a similar effect.

One limitation of our study is that we have not evaluated whether TLR-2 expression is up-regulated in other peripheral blood cells. Sabroe et al [36] have shown that neutrophils and basophils express TLR-2 and TLR-4 albeit at lower levels than monocytes. Of note these authors demonstrated that neutrophils responses to bactetial PAMPs, specifically the up-regulation of CD11b and shedding of L-selectin, were heavily dependent upon the presence of monocytes [36]. These adhesion molecules are up-regulated in neutrophils of COPD patients but not in cells from smokers with normal lung function [37]. Future studies will address, on one hand, the expression of TLR by neutrophils and, on the other hand, the role of monocytes from COPD patients in the response of neutrophils from COPD patients to PAMPs and endogenous ligands.

Another, obvious, limitation of our study is that we analyzed circulating monocytes and not alveolar macrophages. We used this approach because of the difficulties to obtain pulmonary cells during AECOPD and because we decided to start exploring the role of TLRs in COPD using the less invasive technique possible. While this manuscript was under revision, Droemann et al. [38] reported that alveolar macrophages from COPD patients and smokers express less TLR-2 than never smokers and recently we have obtained similar results (Regueiro and Bengoechea, unpublished findings). Droemann and colleagues also examined TLR-2 expression in peripheral blood monocytes and, in contrast to our results, they did not find a significant increase in TLR-2 expression in monocytes from COPD patients [38]. At present we cannot explain this discrepancy although the patients recruited in our study are more homogeneous in terms of age, smoking story and FEV1 than the ones recruited by Droemann et al [38]. This may explain the differences in terms of SD between our studies when the results obtained using flow cytometry are compared which undoubtedly affect the outcome of the statistical analysis.

Care should be taken to directly extrapolate findings obtained in systemic circulation to the pulmonary compartment and *vice versa*. Monocytes enter into the lungs both constitutively to keep alveolar macrophage homeostasis as well as during lung inflammation. It will be interesting to study the contribution of monocytes overexpressing TLR-2 to the lung inflammation of COPD patients and also the possible effect of lung environment on the expression of TLR-2 after monocytes have been recruited. In this context, a recent study using a mice model of acute lung inflammation [39] shows that inflammatory recruited monocytes up-regulate gene expression of chemokines, TNFα and lysosomal proteases and down-regulate TLR-2 expression. Several studies have reported that alveolar macrophages from COPD patients show also these features [38,40].

Our findings may have some clinical implications. First, we showed that steroids reduce TLR-2 expression *in vitro *(fig 4) and, this may happen also *in vivo *(fig 3) arguing against the recently postulated steroid-resistance of COPD [41]. Second, systemic inflammation is a significant contributor to many of the systemic consequences of COPD, including skeletal muscle dysfunction and cardiovascular disease [42]. The latter may be particularly relevant in this context because TLR's have been implicated in the pathogenesis of atherosclerosis [43]. Thus therapeutic strategies to control TLR-2-dependent signalling might be useful in COPD. However, paralysing the TLR-2 -dependent activation of the innate immunity may increase the risk of bacterial infections. An alternative approach would be to diminish TLR-2 expression. This could be achieved by blocking the effect of IL-6 on TLR-2 expression using an antibody against the receptor of this cytokine [44,45] or blocking the IL-6 intracellular signalling pathway through the induction of SOCS3, an endogenous signalling repressor of cytokine signals [44,46]. *In vitro *studies are currently going on in our laboratory to test the feasibility of these approaches in COPD.

## Conclusion

In summary, our study reveals abnormalities in TLRs expression in peripheral blood monocytes from COPD patients, highlights its potential relationship with systemic inflammation in these patients and identifies potential novel therapeutic targets.

## Competing interests

All author(s) declare that they have no competing interest.

## Authors' contributions

Most of the experiments of this study were done by J Pons, V Regueiro and C Santos. J Ferrer studied the effect of cytokines on Toll-like receptor expression. All the clinical studies were done by J Sauleda and M López. The report was written and edited by J Pons, AGN Agustí and JA Bengoechea. AGN Agustí and JA Bengoechea designed and supervised the project. All authors have read and approved this manuscript.
